# Genome-wide association study and genomic prediction of root system architecture traits in Sorghum (*Sorghum bicolor* (L.) Moench) at the seedling stage

**DOI:** 10.1186/s12870-025-06077-w

**Published:** 2025-01-17

**Authors:** Muluken Enyew, Mulatu Geleta, Kassahun Tesfaye, Amare Seyoum, Tileye Feyissa, Admas Alemu, Cecilia Hammenhag, Anders S. Carlsson

**Affiliations:** 1 Department of Plant Breeding, Swedish University of Agricultural Sciences, Alnarp, Sweden; 2 Institute of Biotechnology, Addis Ababa University, Addis Ababa, Ethiopia; 3 School of Biological Sciences, Washington State University, Pullman, USA; 4 Bio and Emerging Technology Institute, Addis Ababa, Ethiopia; 5 National Sorghum Research Program, Crop Research Department, Melkassa Agricultural Research Center, Ethiopian Institute of Agricultural Research, Adama, Ethiopia; 6 Department of Agronomy, Purdue University, West Lafayette, IN USA

**Keywords:** Genomic prediction, GWAS, Quantitative trait locus, Root system architecture, Sorghum

## Abstract

Root system architecture (RSA) plays an important role in plant adaptation to drought stress. However, the genetic basis of RSA in sorghum has not been adequately elucidated. This study aimed to investigate the genetic bases of RSA traits through genome-wide association studies (GWAS) and determine genomic prediction (GP) accuracy in sorghum landraces at the seedling stage. Phenotypic data for nodal root angle (NRA), number of nodal roots (NNR), nodal root length (NRL), fresh shoot weight (FSW), dry shoot weight (DSW), and leaf area (LA) were collected from 160 sorghum accessions grown in soil-based rhizotrons. The sorghum panel was genotyped with 5,000 single nucleotide polymorphism (SNP) markers for use in the current GWAS and GP studies. A multi-locus model, Fixed and random model Circulating Probability Unification (FarmCPU), was applied for GWAS analysis. For GP, ridge-regression best linear unbiased prediction (RR-BLUP) and five different Bayesian models were applied. A total of 17 SNP loci significantly associated with the studied traits were identified, of which nine are novel loci. Among the traits, the highest number of significant marker-trait associations (MTAs) was identified for nodal root angle on chromosomes 1, 3, 6, and 7. The SNP loci that explain the highest proportion of phenotypic variance (PVE) include *sbi32853830* (PVE = 18.2%), *sbi29954292* (PVE = 18.1%), *sbi24668980* (PVE = 10.8%), *sbi3022983* (PVE = 7%), *sbi29897704* (PVE = 6.4%) and *sbi29897694* (PVE = 5.3%) for the traits NNR, LA, SDW, NRA, NRL and SFW, respectively. The genomic prediction accuracy estimated for the studied traits using five Bayesian models ranged from 0.30 to 0.63 while it ranged from 0.35 to 0.60 when the RR-BLUP model was used. The observed moderate to high prediction accuracy for each trait suggests that genomic selection could be a feasible approach to sorghum RSA-targeted selection and breeding. Overall, the present study provides insights into the genetic bases of RSA and offers an opportunity to speed up breeding for drought-tolerant sorghum varieties.

**Supplementary Information:**

The online version contains supplementary material available at 10.1186/s12870-025-06077-w.

## Introduction

Sorghum (*Sorghum bicolor* (L.) Moench) is an annual C_4_ plant of the *Poaceae* (*Gramineae*) family, classified as a diploid species, with a genome of 732.2 Mega base pairs (Mb) [1]. In addition to being gluten-free and rich in starch and protein for adequate digestibility, it is rich in condensed health beneficial compounds [2, 3]. Sorghum is the fifth most produced cereal crop, following maize, rice, wheat, and barley, with a global production of approximately 57.6 million metric tons [4]. It is one of most suitable crops for future climate change because of its ability to grow under harsh environmental conditions, such as drought, high salinity and high temperature [5].

Despite being generally drought tolerant and predominantly cultivated in dryland areas, drought remains a major challenge for sorghum production [6]. Several studies have been undertaken to investigate the genetic and physiological mechanisms that enhance drought tolerance in sorghum [7–11]. Among different physiological mechanisms, those involving root system architecture (RSA) are crucial for drought adaptation. Root traits, such as angle, number, length, surface area, density, and weight of roots play crucial roles in drought tolerance. These traits determine the soil area available for water and nutrient uptake and help anchor the plant system securely in the soil [12]. They are essential in nutrient and water uptake, resilience to environmental stresses, and overall plant performance. However, they received limited attention during germplasm screening and in breeding programs because root trait phenotyping is labor-intensive and technically demanding. In sorghum, the role of RSA in enhancing plants’ ability to extract water, thereby contributing to increased grain yield under drought-stress conditions has been documented [8, 9]. Therefore, characterizing the genetic basis of RSA traits is crucial to understanding the biological mechanisms governing RSA. This facilitates potential target identification for marker-assisted selection (MAS) in breeding programs. Ultimately, this leads to its enhanced drought tolerance allowing increased and sustainable production.

Genome-wide association studies (GWAS) is a valuable tool to identify favorable alleles associated with desirable traits through the utilization of phenotypic and genotypic variations within a plant species [13]. Through this method, several genomic regions associated with different agronomic traits have previously been identified in many crops, including wheat [14, 15], maize [16], rice [17], barley [18], turnip rape [19] and sorghum [20, 21]. The associations of molecular markers with RSA traits in sorghum have been investigated with both linkage mapping using recombinant inbred lines (RIL) [9, 22, 23] and association mapping using sorghum association panels [24–27]. However, there is a need for further research to shed more light on the genetic factors regulating RSA traits, which are crucial for enhancing sorghum’s resilience to climate change. While GWAS has identified loci associated with RSA traits in sorghum, genomic prediction (GP) offers a promising approach to estimate breeding values for these complex traits based on genomic data alone.

Genomic prediction is an emerging method that uses predictive models trained on a population comprising individuals with both phenotypic and genotypic data to estimate the breeding values of individual plants solely based on their genomic information [28, 29]. This method is particularly valuable for traits like RSA, which are difficult and time-consuming to phenotype. Genomic prediction has been applied in sorghum to predict breeding values for agronomically important traits using different prediction models [30, 31]. However, it has not been applied on sorghum RSA traits to determine their genomic prediction accuracy. Therefore, the objectives of this study were to (1) identify genomic regions associated with RSA traits through GWAS and (2) determine genomic prediction accuracy for RSA traits in sorghum landraces.

## Materials and methods

### Plant materials

Among the 160 accessions, 121 were landrace accessions obtained from the Ethiopian Biodiversity Institute (EBI) representing diverse geographical regions in Ethiopia, 36 landrace accessions were collected from farmers’ fields in drought-prone areas in Ethiopia [32], and 3 accessions were improved sorghum varieties obtained from Melkassa Agricultural Research Center (MARC), Ethiopia. For the sake of simplicity, the accessions are referred to as genotypes from here on.

### Phenotyping of root system architectural and shoot traits

The phenotypic data of RSA traits of sorghum genotypes characterized by Enyew et al. [33] using the soil-based root chamber phenotyping were further explored in this study. Briefly, the soil based-root chamber was built in two transparent perspex sheets with 4 mm thickness, 60 cm height and 80 cm width. In each part of the chamber, two sorghum seeds were planted at a depth of 3 cm with the embryo facing the transparent wall to allow root visibility as described by Enyew et al. [34]. One of the healthier plant was kept to grow following three days of germination. The experiment was done in a controlled greenhouse at a day/night temperature of 28/22°C and an average relative humidity of 70%. The experiment was laid out in a complete randomized design with three replications. Each of the three replicates was planted in three different dates. The phenotypic data for nodal root angles (NRA), number of nodal roots (NNR), nodal root length (NRL), fresh shoot weight (FSW), dry shoot weight (DSW), and leaf area (LA) were collected after 21 days of planting at 5 to 6 leaf stages of the plants. Phenotypic analysis of variance and repeatability (H^2^) for each trait were conducted using R software. The META-R software package, version 6.0 (Alvarado et al., 2020) was used to estimate the Best Linear Unbiased Prediction (BLUP). The raw data and the BLUP values used for the GWAS and GP analysis are provided in Supplementary Table 1.

### Genotyping and genome wide association study

The genotypic data used in the current study were previously published by Enyew et al. [20]. Briefly, genotyping of sorghum genotypes targeting 5000 SNP markers was conducted using SeqSNP, which is an advanced targeted genotyping by sequencing method. All markers were designed in a highly specific assay that prevents off-target hits with the sorghum reference genome, ensuring complete coverage (with two oligo probes used for each target), as detailed in Enyew et al. [35].The targeted SNPs were sequenced using the Illumina NextSeq 500/550 v2 system. The obtained data were filtered to obtain only loci with two alleles (bi-allelic), which resulted in 4,639 SNP markers. Further filtering of the data to obtain loci with minor allele frequency (MAF) > 0.05, heterozygosity < 13% and missing genotypes < 2% resulted in 2,950 high-quality SNPs (Supplementary Table 2).

For GWAS, the Genome Association and Prediction Integrated Tool (GAPIT) R package, version 3.4 [36] was implemented in the R environment, version 4.0.3 [37]. The GWAS was performed using 2,950 SNP markers along with the RSA traits of 160 sorghum genotypes. The pairwise genetic relationship (kinship matrix) was calculated according to VanRaden [38] using the pipeline implemented in GAPIT. Multi-locus GWAS model, FarmCPU was used to perform the marker-trait association (MTA) analysis [39]. The Bonferroni threshold adjusted for multiple marker tests at *P* ≤ 0.05 was implemented to avoid potential false-positive MTAs. Manhattan and Quantile–quantile (QQ) plots were created through the *qqman* R package, Version: 0.1.9 [40]. Q–Q plots of p-values were used to visualize the performance of the GWAS model after accounting for population structure and familial relatedness. The physical map positions of all significantly associated SNPs were used to search and identify candidate genes in the sorghum SNP database SorGSD (http://sorgsd.big.ac.cn) [41], which is linked to the annotation on Phytozome v12.1 (www.phytozome.net) sorghum genome database [42]. The functional annotation of candidate genes, including Gene Ontology (GO) and KEGG pathway annotations were retrieved from the SorGSD database, which is linked to the Phytozome v12.1 sorghum genome database for further pathway and functional insights.

### Genomic prediction and cross-validation analysis

The phenotypic and genotypic datasets used for GWAS were also utilized for genomic prediction (GP). Six different genomic prediction models were evaluated for the studied RSA traits. The rrBLUP package, version 4.6.3 [43] within the R environment was used to implement the RR-BLUP model, fitting the basic linear mixed model:

$${\rm{Y = \beta + Z\mu + \varepsilon }}$$

where *Y* represents the *N* × 1 vector of adjusted phenotypic means (BLUPs) for each of the studied RSA traits. *β* is the intercept, and *Z* is the *N × Nm* matrix of SNP markers where *N* refers to the number of genotypes, and *Nm* represents the number of SNP markers. The random SNP effects (*µ*), represented as the *Nm* × 1 vector, were obtained using the “mixed.solve” function, assuming *µ~N*(0, I), where *I* is the identity matrix, and *µ* represents the genetic variance contributed by each SNP and *ε* is the *N* × 1 vector of residual effects.

Five Bayesian-based models from the BGLR package, version1.1.3 [44] were used to further evaluate the predictability of the studied RAS traits. These models vary in how they handle marker effects, with most assuming unequal genetic variance across chromosomes to account for major QTL effects. Different prior assumptions in these models influence the type of shrinkage or variable selection applied to marker effect estimates. The Bayesian ridge regression (BRR) model uses a Gaussian prior, shrinking marker effects uniformly. BayesA [45] and Bayesian LASSO (BL) models use priors (scaled-t and Laplace) with more mass at zero and thicker tails, resulting in effect-size-dependent shrinkage [46]. BayesC and BayesB models apply finite mixture priors: BayesC uses a mix of a point mass at zero and a Gaussian slab, while BayesB uses a mix of a point mass at zero and a scaled-t slab [45].

All BGLR analyses were conducted with a Markov Chain Monte Carlo sampler for 12,000 iterations, with a thinning interval of 10 and a burn-in of 2,000 iterations.

The accuracy of GP was determined by using cross-validation, where 80% of the genotypes were randomly selected for a training set while the remaining 20% were used as a test set. The cross-validation analysis was repeated 500 times for the RR-BLUP models and the five Bayesian models. The predictive abilities of models were evaluated by examining the correlation between the GEBVs of individuals in the test set and their BLUP values derived from the phenotypic data. The prediction accuracy was calculated by dividing the predictive ability by the square root of the broad-sense heritability of the traits, as described by previous studies Legarra et al. and Alemu et al. [47, 48].

## Results

### Phenotypic variation and heritability

**Fig. 1:**
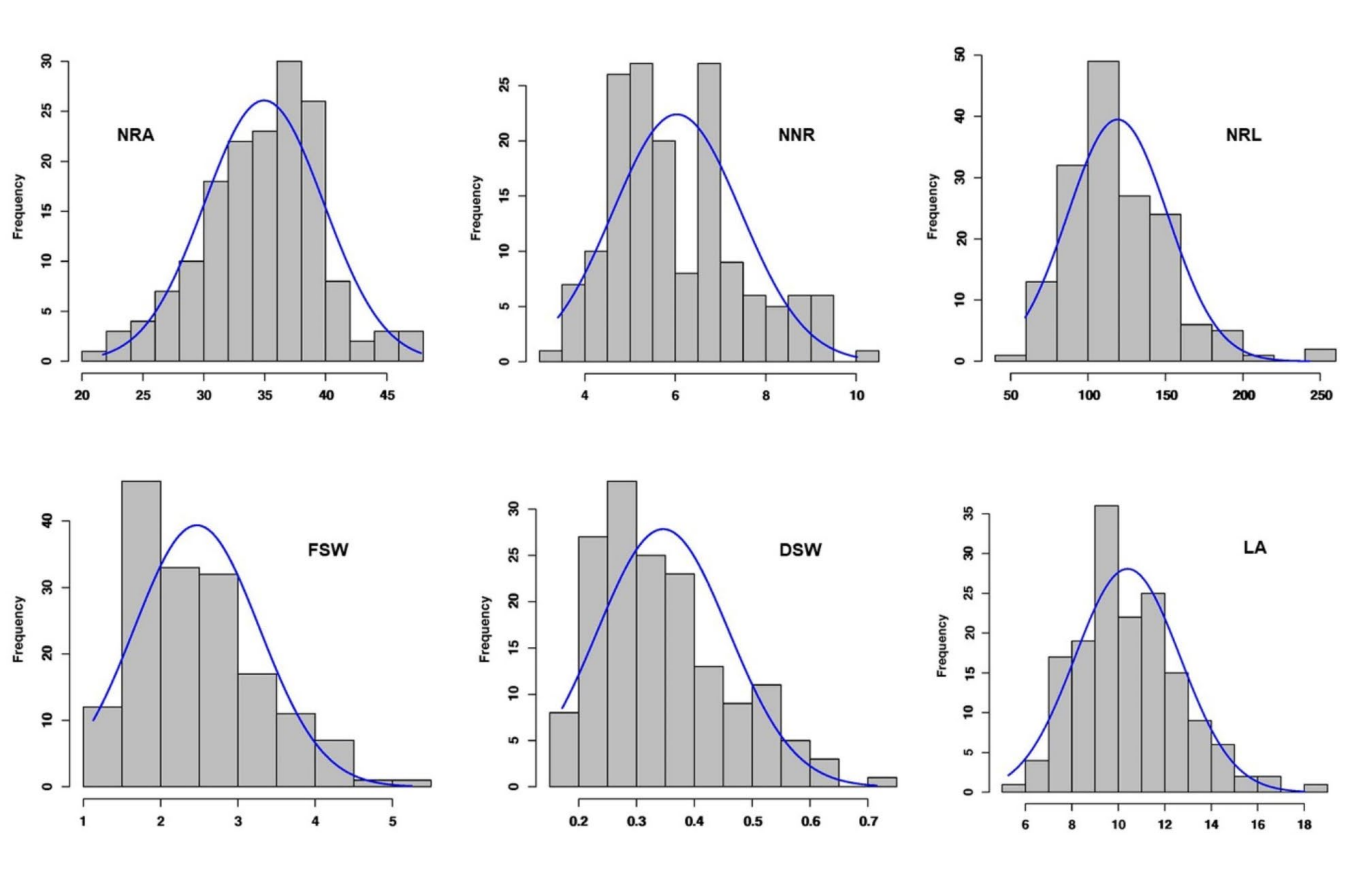
Histogram of the frequency distributions of Best Linear Unbiased Prediction (BLUP) values of six RSA traits targeted in this study. NRA = nodal root angle, NNR = number of nodal roots, NRL = nodal root length, FSW = fresh shoot weight, DSW = dry shoot weight, and LA = leaf area

The analysis of variance (ANOVA) revealed highly significant (*p* < 0.001) variation among genotypes for all studied RSA and shoot traits (Supplementary Table 3). The phenotypic variation of traits appeared to be normally distributed (Fig. 1). The repeatability (H²) of the RSA traits was high for LA (61%), NRA (63%), DSW (70%), FSW (74%), and NNR (85%), except for NRL (48.4%), which was moderate (Supplementary Table 3).

### Identification of candidate genes for root system architecture and shoot traits in sorghum via genome-wide association studies

**Fig. 2:**
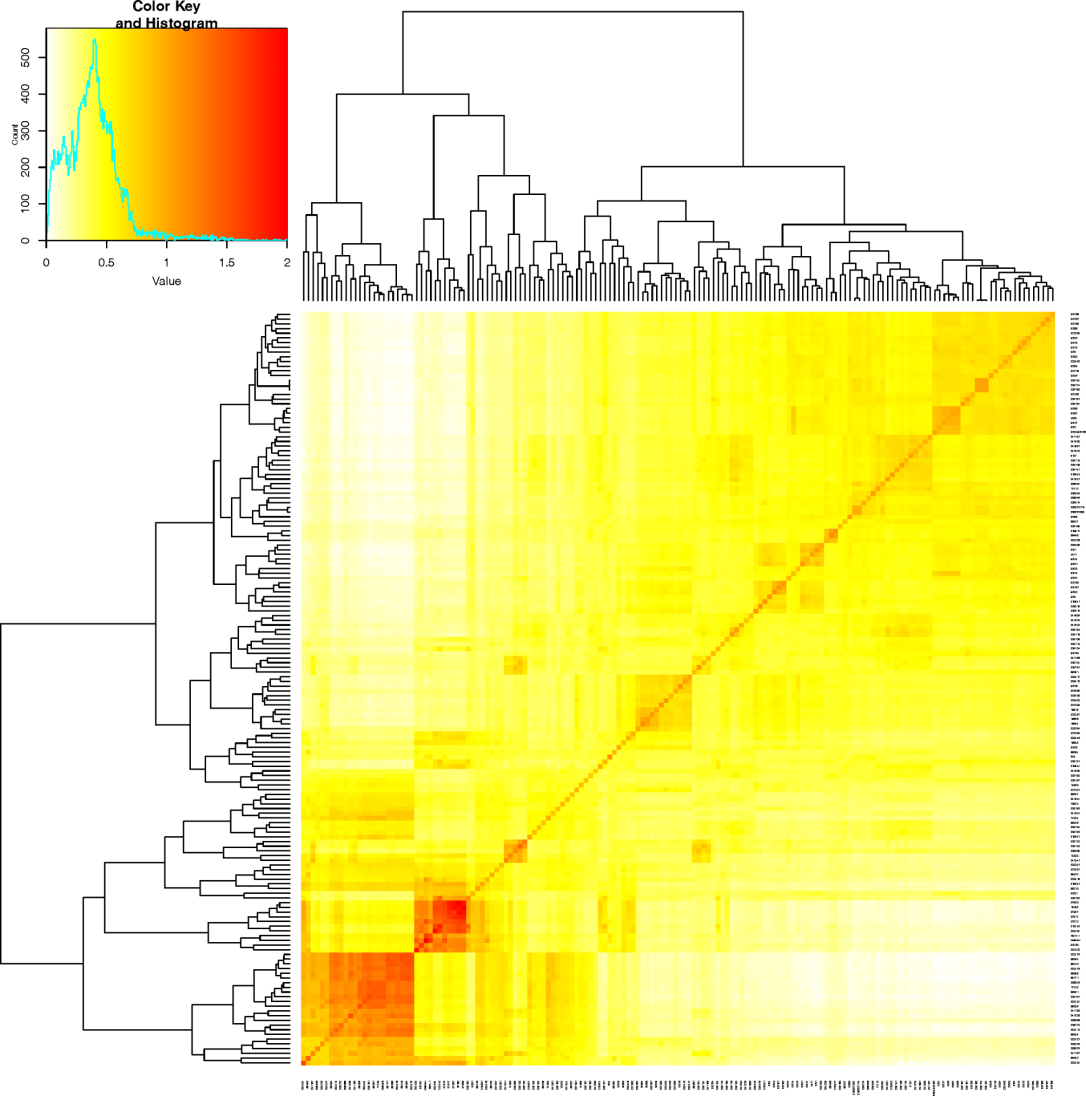
A kinship matrix presented as a heatmap, with red representing the highest correlation between genotype pairs and yellow indicating the lowest correlation. A hierarchical tree of individuals is shown based on their kinship relationships

A genome-wide association study (GWAS) was performed to identify genetic loci associated with the six RSA traits evaluated in the diverse sorghum panel. A total of 2,950 high-quality SNP markers were used to estimate kinship within the panel using the VanRaden method. This method produces a matrix with values ranging from 0 to 2, where “0” indicates no genetic relatedness and “2” shows complete genetic relatedness (individuals are genetically identical) (Fig. 2). The distribution of the coefficients from the kinship analysis of the 160 genotypes shows weak genetic relatedness within the panel.

The Bonferroni threshold for multiple markers test with 5% probability of type I error was estimated as 0.05/2950 = 1.69 × 10^− 5^. The whole list of identified SNP loci associated with studied traits above the Bonferroni threshold is presented in Table 1 and graphically displayed in Manhattan plots (Fig. 1). In total, 17 SNP loci were identified for the studied traits (Table 1) and the favorable allele distribution of significant SNPs across accessions is provided in Supplementary Table 4. Only a single SNP marker was identified associated with two different traits (Table 1). Quantile–Quantile plots indicated an exact alignment between the expected and observed -log10 p-values under the null hypothesis at the start of the plot (Fig. 1). Toward the right end, there is a deviation of observed values from the null hypothesis, suggesting a true positive association between the SNPs and the traits (Fig. 1). Thus, the GWAS model used in this study effectively controls the cofounding effects, making the results reliable and reducing the likelihood of reporting false negatives. The candidate genes comprising the SNPs showing a significant association with studied traits were identified and their putative functions were characterized by searching the map position of the significant SNPs in sorghum SNP database (SorGSD) (Table 2).

### Genetic dissection of the root system architecture traits

**Fig. 3:**
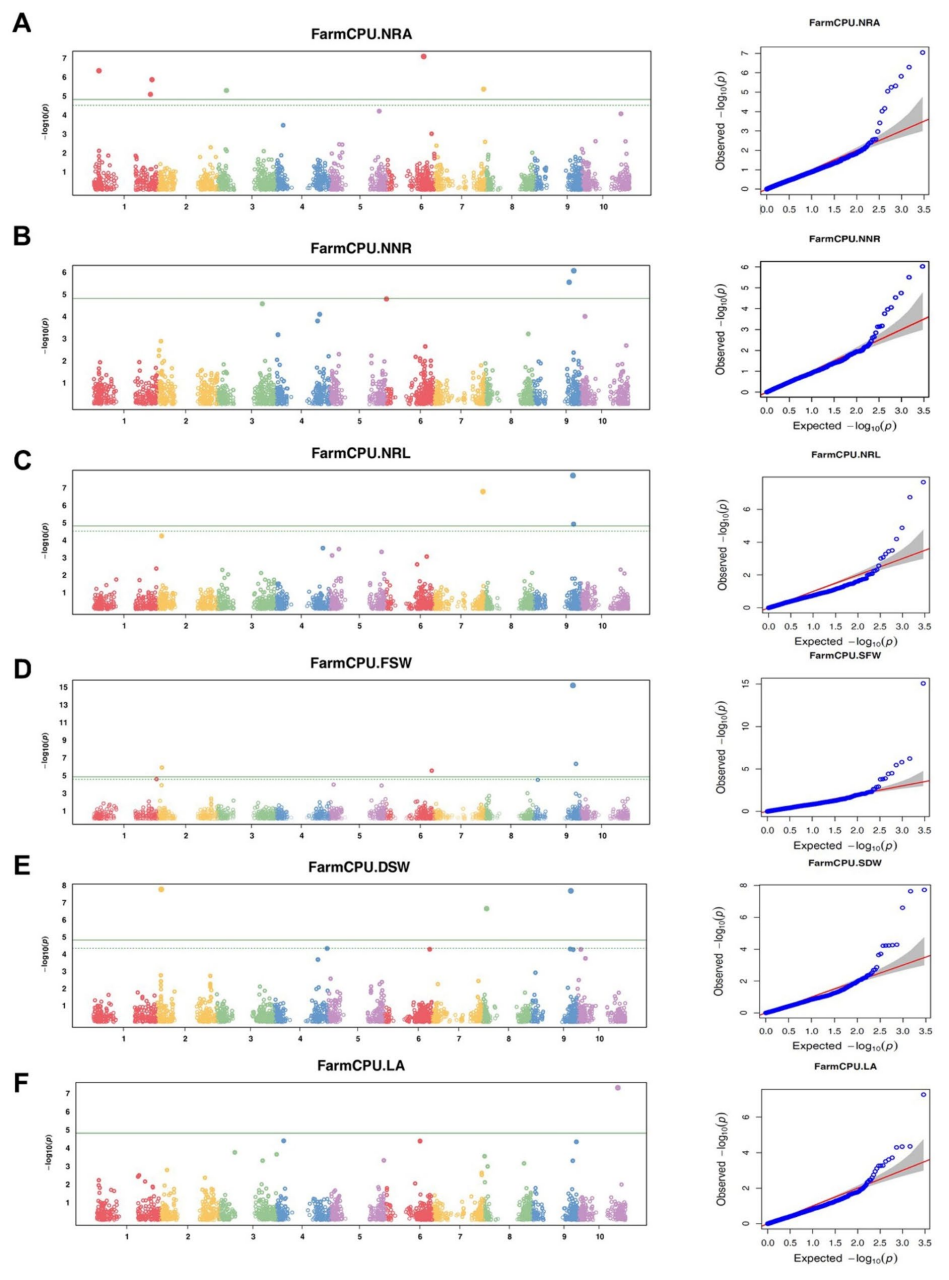
The Manhattan and Quantile–quantile (QQ) plots showing the significant SNPs across the 10 sorghum chromosomes identified by the current GWAS analysis for (**A**) the nodal root angle (NRA), (**B**) number of nodal roots (NNR), (**C**) nodal root length (NRL), (**D**) fresh shoot weight (FSW), (**E**) dry shoot weight (DSW) and (**F**) leaf area (LA) at seedling stage

**Fig. 4:**
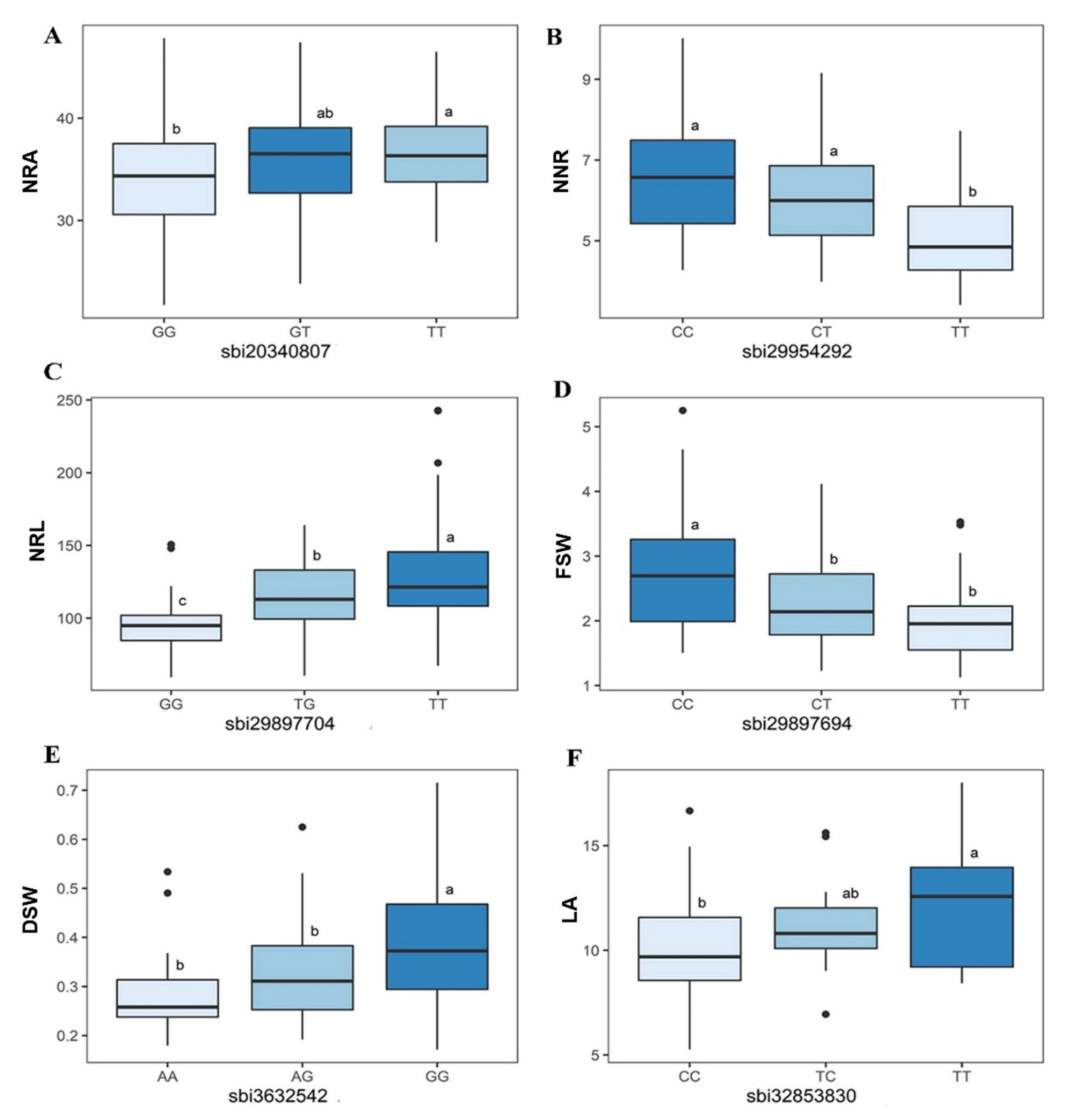
Boxplots of the most significant SNPs *sbi20340807, sbi29954292, sbi29897704, sbi29897694, sbi3632542 and sbi32853830* with their allelic effects on nodal root angle (NRA), number of nodal roots (NNR), nodal root length (NRL), fresh shoot weight (FSW), dry shoot weight (DSW), and leaf area (LA), respectively. Statistical significance for differences between allele effects was determined using Tukey’s HSD (honestly significant difference) test. Different letters in the same box indicate significant phenotypic differences among plants with corresponding genotypes at that locus (*P* < 0.05)

In the present study, the highest number of significant marker-trait associations was identified for nodal root angle. In total, five SNP loci were identified significantly associated with nodal root angle on chromosomes 1, 3, 6, and 7 (Table 1and Fig. 3). Two of these SNP markers, *sbi3022983* and *sbi7781202* on chromosomes 1 and 3 had the highest percentage of explained phenotypic variance with 6.97 and 5.35%, respectively (Table 1). The effects of the marker *sbi20340807* alleles on nodal root angle significantly grouped studied sorghum genotype (*P* < 9.04 × 10^− 8^ (Fig. 4).

All identified SNP markers significantly associated with nodal root angle were located within different genes (Table 2). Two SNP loci, *sbi3022983* and *sbi7781202* that had the largest effects on nodal root angle, on chromosome 1 at 73.7 Mb (PVE = 6.87%) and on chromosome 3 at 11 Mb (PVE = 5.35%) (Table 1) were found within the genes, *Sobic.001G462500* and *Sobic.003G121200*, respectively. The gene *Sobic.001G462500* encodes natural resistance-associated macrophage that play transporting a wide range of divalent metal ions while *Sobic.003G121200* encodes *PPR* repeat containing protein that plays a key role in physiological processes contributing to plant growth and development. Among the markers significantly associated with nodal root angle, sbi20340807 on chromosome 6 (47.6 Mb) is the most significant (*P* < 9.04 × 10^− 8^), and is located within the gene *Sobic.006G106200* which encodes a *NAP* domain containing protein. This protein plays a major role in regulating leaf senescence. Additionally, the *Sobic.007G193200* gene was identified close to a SNP marker significantly associated with nodal root length. This gene encodes *MTA*/*SAH* nucleosidase, a crucial metabolite involved in biosynthetic pathways and the biosynthesis of ethylene and polyamines, which play critical roles in plant physiology.

The GWAS analyses detected two significantly associated SNPs with NNR (Table 1and Fig. 3). These SNP markers, *sbi29649877* and *sbi29954292* were identified on chromosome 9 at positions 45.2 Mb and 51.1 Mb, respectively. *Sbi29954292* accounted for the highest phenotypic variation (18.07%) while *sbi29649877* explained 2.37% of the phenotypic variation in NNR. These markers are located within the coding sequences of the genes *Sobic.009G112500* (encoding lipase) and *Sobic.009G154800* (encoding syntaxin 6, N-terminal domain containing protein), respectively (Table 2).

GWAS identified two SNPs significantly associated with nodal root length (Table 1). These SNPs are located on chromosomes 9. The *sbi29939092* marker, on chromosome 9, explained the highest phenotypic variation (6.4%) for NRL was located 46 kb upstream of the gene *Sobic.009G153101* that encodes zinc finger, *C3HC4* type domain containing protein. The other SNP marker *sbi29897704* (50.1 Mb) is located within a gene *Sobic.009G143700. Sobic.009G143700* encodes *NAC103* regulates *ABA* response during seed germination and seedling growth in Arabidopsis (Table 2).

### Genetic control of shoot fresh and dry weight and leaf area

In this study, four SNP markers significantly associated with FSW were identified on chromosomes 2, 6, and 9 (Table 1). The most significant (*P* < 8.25 × 10^− 16^) marker *sbi29897694* (50.1 Mb), on chromosome 9, explained 5.3% of the total phenotypic variation in FSW. This SNP marker is located within the *Sobic.009G143700* gene that encodes no apical meristem protein, which plays a role in plant development and is critical for proper leaf and flower patterning. The other identified genes linked to FSW-associated SNP markers were *Sobic.002G063600* and *Sobic.009G185700. Sobic.002G063600* encodes Leucine Rich Repeat family protein while *Sobic.009G185700* encodes mutS domain V family protein, which are known for their roles in cell wall developmental processes and DNA repair and recombination (Table 2).

The GWAS analyses detected three SNP markers significantly associated with DSW (Table 1). These three SNPs, *sbi3632542*, *sbi24668980* and *sbi29939008* located on chromosomes 2, 8 and 9 explained 4.25, 10.8, and 7.51% of the total phenotypic variance respectively. *Sobic.002G063600* (Leucine Rich Repeat family protein), *Sobic.008G050800* (Uncharacterized protein), and *Sobic.009G152400* (encoding glutamine cyclotransferase precursor) genes were associated with these SNP markers in that order (Table 2).

A single SNP marker was significantly associated with LA (Table 1). This marker *sbi32853830* is located on chromosome 10 at position 51.5 Mb, which accounted for a phenotypic variation of 18.22%. The SNP is within the gene *Sobic.010G176800* encoding *ABC* transporter and ATP-binding protein (Table 2). This gene plays a critical role in most aspects of cell physiology, including nutrient uptake and energy generation.

**Table 1:** List of SNPs significantly associated with the studied traits and their descriptions

Trait	SNP ID	Alleles	Chr	Position	MAF	*P*-value	Effect	PVE
NRA	*sbi7781202*	A/G	3	11,053,605	0.14	5.64 × 10^− 6^	2.20	5.35
	*sbi20340807*	G/T	6	47,614,312	0.38	9.04 × 10^− 8^	2.26	1.84
	*sbi24257982*	G/C	7	62,580,069	0.09	1.44 × 10^− 6^	3.43	4.66
	*sbi2946469*	C/A	1	71,747,223	0.29	8.97 × 10^− 6^	2.00	2.21
	*sbi3022983*	G/A	1	73,690,076	0.09	1.52 × 10^− 6^	3.80	6.97
NNR	*sbi29649877*	C/G	9	45,256,800	0.36	3.12 × 10^− 6^	-0.48	2.37
	*sbi29954292*	C/T	9	51,147,626	0.41	9.45 × 10^− 7^	-0.61	18.07
NRL	*sbi29897704*	T/G	9	50,101,376	0.31	2.20 × 10^− 8^	18.47	1.66
	*sbi29939092*	G/C	9	50,893,174	0.36	1.34 × 10^− 5^	13.31	6.40
FSW	*sbi3632542*	A/G	2	6,203,646	0.38	1.56 × 10^− 6^	0.34	3.87
	*sbi29897694*	C/T	9	50,101,247	0.32	8.25 × 10^− 16^	-0.74	5.28
	*sbi30071808*	C/T	9	53,885,817	0.21	6.00 × 10^− 7^	0.31	0.88
	*sbi20839691*	G/A	6	58,860,779	0.21	3.50 × 10^− 6^	0.43	1.54
DSW	*sbi24668980*	G/A	8	5,053,406	0.08	2.24 × 10^− 7^	0.09	10.76
	*sbi3632542*	A/G	2	6,203,646	0.37	1.92 × 10^− 8^	0.05	4.25
	*sbi29939008*	C/T	9	50,892,152	0.31	2.33 × 10^− 8^	-0.06	7.51
LA	*sbi32853830*	T/C	10	51,473,805	0.17	5.47 × 10^− 8^	1.10	18.22

NRA = nodal root angle, NNR = number of nodal roots, NRL = nodal root length, FSW = fresh shoot weight, DSW = dry shoot weight, and LA = leaf area. Chr = Chromosome, PVE = Proportion of phenotypic variance, MAF = Minor allele frequency, Favorable allele highlighted in bold

**Table 2:** Descriptions of candidate genes associated with significant marker-trait associations, including their annotated functions

Trait	SNP ID	Allele	Chr	Position	Gene name	Candidate gene description
NRA	*sbi2946469*	C/A	1	71,747,223	*Sobic.001G439400*	glycosyl hydrolase family 10 protein
	*sbi3022983*	G/A	1	73,690,076	*Sobic.001G462500*	natural resistance-associated macrophage
	*sbi7781202*	A/G	3	11,053,605	*Sobic.003G121200*	PPR repeat containing protein
	*sbi20340807*	G/T	6	47,614,312	*Sobic.006G106200*	NAP domain containing protein
	*sbi24257982*	G/C	7	62,580,069	*Sobic.007G193200*	MTA/SAH nucleosidase
NNR	*sbi29649877*	C/G	9	45,256,800	*Sobic.009G112500*	lipase
	*sbi29954292*	C/T	9	51,147,626	*Sobic.009G154800*	syntaxin 6, N-terminal domain containing protein
NRL	*sbi29897704*	T/G	9	50,101,376	*Sobic.009G143700*	NAC103
	*sbi29939092*	G/C	9	50,893,174	*Sobic.009G153101*	zinc finger, C3HC4 type domain containing protein
FSW	*sbi3632542*	A/G	2	6,203,646	*Sobic.002G063600*	Leucine Rich Repeat family protein
	*sbi20839691*	G/A	6	58,860,779	*Sobic.006G249200*	Uncharacterized protein
	*sbi29897694*	C/T	9	50,101,247	*Sobic.009G143700*	no apical meristem protein
	*sbi30071808*	C/T	9	53,885,817	Sobic.009G185700	mutS domain V family protein
DSW	*sbi3632542*	A/G	2	5,053,406	*Sobic.002G063600*	Leucine Rich Repeat family protein
	*sbi24668980*	G/A	8	6,203,646	*Sobic.008G050800*	Uncharacterized protein
	*sbi29939008*	C/T	9	50,892,152	*Sobic.009G152400*	glutamine cyclotransferase precursor
LA	*sbi32853830*	T/C	10	51,473,805	*Sobic.010G176800*	ABC transporter, ATP-binding protein, putative

NRA = nodal root angle, NNR = number of nodal roots, NRL = nodal root length, FSW = fresh shoot weight, DSW = dry shoot weight, LA = leaf area, Chr = Chromosome

### Genomic prediction of root system architecture traits

**Fig. 5:**
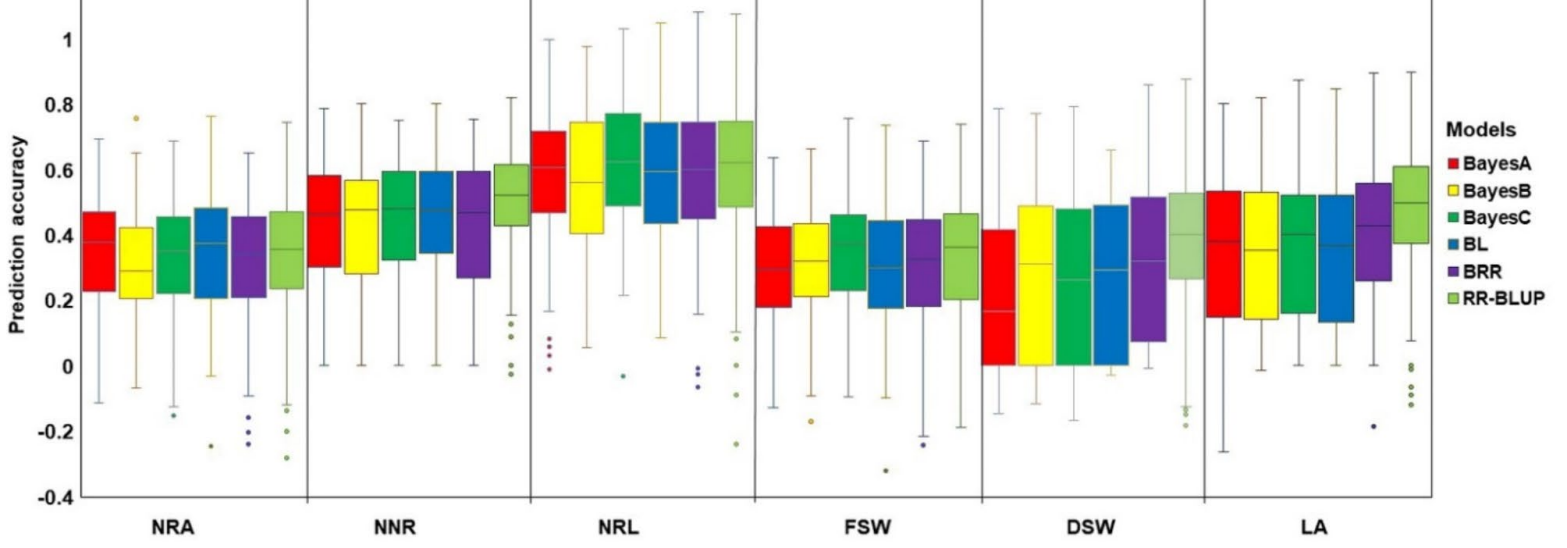
The genomic prediction (GP) accuracy of five Bayesian models and the Ridge-regression best linear unbiased prediction (RR-BLUP) model for the nodal root angle (NRA), number of nodal roots (NNR), nodal root length (NRL), fresh shoot weight (FSW), dry shoot weight (DSW), and leaf area (LA) in sorghum at seedling stage

Genomic prediction for the studied traits was conducted via 80–20% training-test set cross-validation analysis with six different prediction models (Fig. 5 and Supplementary Table 5). The five Bayesian models and the RR-BLUP model produced genomic estimated breeding values (GEBVs) with small differences in prediction accuracy across all traits (Fig. 5 and Supplementary Table 5). The prediction accuracy with the five Bayesian models ranged from 0.30 to 0.63 while with the RR-BLUP model it ranged from 0.34 to 0.60 across the studied traits. The traits with the lowest and highest prediction accuracy were FSW and NRL as revealed by both the RR-BLUP and Bayesian models (Fig. 5and Supplementary Table 5).

## Discussion

### Genetic control of root system architecture traits

Crop tolerance to abiotic stresses has been achieved through improving shoot and root traits, thereby increasing agricultural productivity [5, 49]. The RSA traits are among the most important traits for extracting water and nutrients deep in the soil that help the plant adapt to harsh environmental conditions [6, 8, 9]. A deep and comprehensive understanding of the genetic basis of RSA traits could aid in enhancing the root systems of sorghum varieties under water and/or nutrient stress conditions. GWAS provides opportunities to understand the genetic basis of complex quantitative traits such as RSA by analyzing high-throughput phenotypic and genotypic data. In the present study, novel and previously reported genomic regions associated with RSA traits were identified. Previously reported loci associated with RSA traits in sorghum [9, 22–27, 50, 51] are summarized in Supplementary Table 6. The differences between the genomic regions detected in the present study and those identified in previous studies are discussed in more detail below.

Genomic regions associated with nodal root angle in sorghum have been previously reported [24, 26]. In this study, five SNP loci significantly associated with nodal root angle were identified on chromosomes 1, 3, 6, and 7. Among these loci, three were co-located with previously reported QTL regions. For instance, a SNP locus on chromosome 6 (*sbi20340807*) at position 47.6 Mb is co-located with QTL regions identified in previous studies on Ethiopian sorghum landraces [24, 26]. This SNP is a missense mutation within a gene *Sobic.006G106200*, which encodes NAP domain containing protein and plays an important role in regulating leaf senescence by promoting chlorophyll degradation through ABA Biosynthesis [52]. Transgenic plants having an altered level of NAP protein display delayed leaf senescence relative to a non-transgenic plant and improved yield [53]. The delay in leaf senescence (stay-green) is associated with increased water availability, possibly via greater water and nutrient absorption through RSA traits. Genotypes with narrow root angles displayed higher drought tolerance and stay-green properties [9] and improved yield in sorghum [8]. This is due to the fact that plants with deeper roots and narrow root angles extract water and nitrogen more effectively [9, 54–56]. Therefore, *Sobic.006G106200* could be a gene involved in controlling the nodal root angel variation explained by the marker *sbi20340807* in this study.

Lopez et al. [22] identified a QTL for nodal root angle on chromosome 3 at the physical position 4.6 Mb using a bi-parental mapping population and this QTL is located about 6.4 Mb away from the SNP locus *sbi7781202* identified on the same chromosome at 11.0 Mb in the present study. This marker is within the *Sobic.003G121200* gene and it explained 5.35% of the total phenotypic variation. The gene encodes a *PPR* repeat containing protein that plays a key role in physiological processes contributing to plant growth and development. The marker *sbi2393610* at position 62.6 Mb on chromosome 7 is located about 8.7 Mb away from the previously detected locus for nodal root angle [57]. This SNP is located within the *Sobic.007G193200* gene encoding MTA/SAH nucleosidase, crucial metabolites involved in the biosynthesis of ethylene and polyamines, which play critical roles in plant physiology. The remaining two loci located on chromosome 1 (*sbi2946469* and *sbi3022983*) are likely to be novel loci associated with the nodal root angle of sorghum. The SNP marker, *sbi3022983* on chromosome 1 at 73.7 Mb was located within the *sobic.001G462500* gene encoding a natural resistance-associated macrophage that plays a role in transporting a wide range of divalent metal ions.

Previous association mapping studies identified marker trait associations for root number in sorghum on chromosomes 1, 2, 4, 6, 7, 8 and 9 [23, 24, 26]. In the present study, the two SNP loci *sbi29649877* and *sbi29954292* significantly associated with nodal root number are located at positions 45.2 Mb and 51.1 Mb, respectively, on chromosome 9, which are in close proximity with a root number associated locus reported by Menamo et al. [26]. The *Sbi29954292* locus explained the highest phenotypic variation (18.07% of the total variation) among the significant markers identified in this study. This SNP is located within the coding sequence of the gene *Sobic.009G112500* that encodes lipase. Whereas, *sbi29649877* is located within the *Sobic.009G154800* gene, which encodes syntaxin 6, N-terminal domain-containing protein.

In the present study, GWAS identified two SNPs that were significantly associated with NRL. Previous association mapping studies on sorghum detected quantitative trait loci (QTLs) for root length on chromosomes 1, 2, 3, 4, 5, 6 and 9 [24–27]. However, none of them are located close to the SNP loci on chromosomes 9 significantly associated with NRL in the present study. The *sbi29939092* locus is located near the upstream gene, *Sobic.009G153101* that encodes zinc finger C3HC4 type domain-containing protein. The C3HC4 zinc finger proteins have been well studied in Arabidopsis reporting their role in various abiotic stresses, such as drought, salt, cold and heat [58–61]. Besides their role in various abiotic stresses, they also function in the development and signaling processes linked to various stress processes like light perception, and peroxisome formation during root and seed development [62, 63]. The other locus, *sbi29897704* (50.1 Mb) on chromosome 9 is located within the genes *Sobic.009G143700*. The gene *Sobic.009G143700* encodes NAC103 that regulates ABA response during seed germination and seedling growth in Arabidopsis.

### Genetic control of shoot fresh and dry weight and leaf area

In the present study, four SNP markers significantly associated with FSW were identified on chromosomes 2, 6, and 9. Among these loci, only the *sbi20839691* locus on chromosome 6 at position 58.9 Mb is located close to a previously reported QTL (at position 57.2 Mb) associated with FSW [50]. The other three markers appeared to be novel markers associated with FSW. The SNP marker *sbi29897694* (*P* < 8.25 × 10^− 16^) on chromosome 9 at position 50.1 Mb is located within *Sobic.009G143700*, a gene that encodes no apical meristem protein and plays a role in plant development and is critical for proper leaf and flower patterning [64–66]. The remaining two identified genes, *Sobic.002G063600* (encoding Leucine Rich Repeat family protein), and *Sobic.009G185700* (encoding mutS domain V family protein), are known for their role in cell wall developmental processes and roles in DNA repair and recombination.

Among the three SNP markers that were significantly associated with DSW in the present study, two (*sbi3632542* and *sbi29939008*) are located in close proximity with previously reported genomic regions on chromosomes 2 and 9, respectively, in sorghum [27]. They are located within the genes *Sobic.002G063600* (Leucine Rich Repeat family protein), and *Sobic.009G152400* (encoding glutamine cyclotransferase precursor), respectively. The third one, *sbi24668980*, is a novel SNP locus identified on chromosome 8, which explained 10.76% of the total phenotypic variance of DSW. It is located within the *Sobic.008G050800* gene that encodes a protein with no currently known function.

In the present study, only one novel SNP marker was significantly associated with LA. Previous association mapping studies in sorghum detected QTLs for LA on all chromosomes except chromosomes 3, 5 and 10 [9, 25, 51]. The significant SNP identified in this study, *sbi32853830*, is located on chromosome 10 at position 51.5 Mb. It is associated with a major QTL, which accounted for 18.2% of the total phenotypic variation in LA. This SNP is located within a gene *Sobic.010G176800* encoding ABC transporter and ATP-binding protein. *Sobic.010G176800* plays a critical role in most aspects of cell physiology, including the uptake of nutrients and energy generation. Additionally, it was reported to be essential for the retention of leaf water in wild barley and rice [67]. Therefore, this locus could be a novel locus that control leaf area variation in sorghum.

### Genomic prediction of root system architecture traits

Genomic prediction is an effective technique for speeding up genetic gains in plant breeding [68]. It estimates the breeding values of individuals for traits of interest by considering all contributing QTLs based on their comprehensive marker information [45]. This method is particularly valuable for developing varieties with desirable traits, such as root system architecture that are multigenic and difficult to measure. The usefulness of genomic prediction in sorghum has been investigated in several studies [30, 31]. However, there were no genomic prediction studies on sorghum RSA traits.

In the present study, the genomic estimated breeding values of 20% of 160 sorghum genotypes for RSA traits were estimated through five Bayesian models and RR-BLUP model. The tested models predicted the genomic estimated breeding values with similar prediction accuracy for all traits. This is in agreement with previous studies that have reported similar prediction accuracy [69–72]. The prediction accuracy of the five Bayesian models ranged from 0.30 to 0.63 while that of the RR-BLUP model ranged from 0.34 to 0.60 across the studied traits. These values are similar to previously reported accuracy for RSA traits in other crops [28, 73, 74]. The lowest prediction accuracy with both the Bayesian models and the RR-BLUP model was observed in FSW, while the highest was in NRL. Given the moderate to high prediction accuracy and the challenges associated with phenotyping RSA traits, genomic selection may be a viable approach for breeding sorghum to improve these traits.

The present study was conducted using 160 sorghum genotypes grown in a controlled greenhouse environment, with 2,950 SNPs selected after quality control. While the study provides valuable insights into the genetic architecture of RSA-related traits in sorghum, it has some limitations. Root phenotyping presents challenges both in controlled environments and in the field, and this study did not include multiple environments, locations, or seasons. We acknowledge the importance of these factors in incorporating environmental influences on RSA traits. In future research, we plan to include accessions from multiple regions and countries to ensure greater genetic diversity and enhance the applicability of our findings. Additionally, we aim to explore gene-environment interactions to better understand how environmental factors impact RSA trait expression. Validating the identified candidate genes in future studies will also be a priority. Finally, expanding the number of genotypes and utilizing denser marker sets will improve the resolution of our findings and the accuracy of trait association studies.

## Conclusion

In this study, both novel and previously reported loci significantly associated with RSA traits were identified in the Ethiopian sorghum genotype at the seedling stage. The majority of the SNPs were located within candidate genes that have key roles in essential biological functions many of which contribute to drought-stress tolerance. These findings offer valuable genetic insights that could aid the development of sorghum cultivars better equipped to withstand water-limited environments. Additionally, the genomic prediction analysis using five Bayesian models and the RR-BLUP model demonstrated small differences in prediction accuracy across all traits. The moderate to high prediction accuracies observed reinforce the potential of genomic selection as an effective strategy for selecting and improving sorghum with desirable RSA traits.

## Electronic supplementary material

Below is the link to the electronic supplementary material.

Supplementary Material 1: Supplementary Table 1: Phenotypic mean values of RSA traits measured for 21-day-old seedlings in Ethiopian sorghum genotypes.

Supplementary Material 2: Supplementary Table 2: List of SNPs, their alleles and chromosomes used for GWAS and genomic prediction in Ethiopian sorghum genotypes.

Supplementary Material 3: Supplementary Table 3: Analysis of variance and heritability for the root system architecture (RSA) traits of 160 sorghum genotypes.

Supplementary Material 4: Supplementary Table 4: The distribution of favorable alleles of significant SNPs across 160 sorghum genotypes.

Supplementary Material 5: Supplementary Table 5: Genomic prediction accuracy (GP) of the five Bayesian models and the Ridge-regression best linear unbiased prediction (RR-BLUP) models.

Supplementary Material 6: Supplementary Table 6: Summary of previously identified SNPs associated with the root system architecture (RSA) traits.

## Data Availability

The data sets supporting the results of this article are included in this manuscript and its supplementary information files. The passport data of the accessions can be found online at: https://link.springer.com/article/10.1007/s11104-023-06373-0: Supplementary file1. All raw sequences are available in the NCBI Sequence Reads Archive (SRA) database under BioProject PRJNA780262”.

## Abbreviations

DSW: Dry shoot weight
EBI: Ethiopian Biodiversity Institute
FarmCPU: Fixed and random model Circulating Probability Unification
FSW: Fresh shoot weight
GP: Genomic prediction
GWAS: Genome-wide association studies
LA: Leaf area
MARC: Melkassa Agricultural Research Center
NNR: Number of nodal roots
NRA: Nodal root angle
NRL: Nodal root length
PVE: Proportion of phenotypic variance
QTLs: Quantitative trait loci
RR-BLUP: Ridge-regression best linear unbiased prediction
RSA: Root system architecture
