# Supplementary material for: Genome-wide association study and genomic prediction of root system architecture traits in Sorghum (Sorghum bicolor (L.) Moench) at the seedling stage
Source: BMC Plant Biol. 2025 Jan 17;25:69. doi: 10.1186/s12870-025-06077-w (PMC11740658; doi:10.1186/s12870-025-06077-w)
Supplement: Supplementary file 3 — Supplementary Material 3: Supplementary Table 3: Analysis of variance and heritability for the root system architecture (RSA) traits of 160 sorghum genotypes. [file 12870_2025_6077_MOESM3_ESM.docx]

**Supplementary Table 3.** Analysis of variance and heritability for the root system architecture and shoot traits of 160 sorghum genotypes.

| **Source of varation** | **NRA** | **NNR** | **NRL** | **FSW** | **DSW** | **LA** |
| --- | --- | --- | --- | --- | --- | --- |
| **Genotype** | 140.90*** | 7.96*** | 6413.00*** | 3.00*** | 0.06*** | 30.40*** |
| **Residuals** | 52.00 | 1.20 | 3311.00 | 0.80 | 0.02 | 11.70 |
| **H^2^** | 63% | 85% | 48% | 74% | 70% | 61% |

NRA = nodal root angle, NNR = number of nodal roots, NRL= nodal root length, FSW = fresh shoot weight, DSW = dry shoot weight, and LA = leaf area, H^2^ = Repeatability
