# Supplementary material for: Genome-wide association study and genomic prediction of root system architecture traits in Sorghum (Sorghum bicolor (L.) Moench) at the seedling stage
Source: BMC Plant Biol. 2025 Jan 17;25:69. doi: 10.1186/s12870-025-06077-w (PMC11740658; doi:10.1186/s12870-025-06077-w)
Supplement: Supplementary file 5 — Supplementary Material 5: Supplementary Table 5: Genomic prediction accuracy (GP) of the five Bayesian models and the Ridge-regression best linear unbiased prediction (RR-BLUP) models. [file 12870_2025_6077_MOESM5_ESM.docx]

**Supplementary Table 5.** Genomic prediction accuracy (GP) of the five Bayesian models and the Ridge-regression best linear unbiased prediction (RR-BLUP) models for (A) the nodal root angles (NRA), (B) number of nodal roots (NNR), (C) nodal root length (NRL), (D) fresh shoot weight (FSW), (E) dry shoot weight (DSW), and (F) leaf area (LA) in sorghum at seedling stage.

| **Traits** | **BayesA** | **BayesB** | **BayesC** | **BL** | **BRR** | **RR-BLUP** |
| --- | --- | --- | --- | --- | --- | --- |
| **NRA** | 0.35 | 0.30 | 0.33 | 0.34 | 0.31 | 0.35 |
| **NNR** | 0.51 | 0.49 | 0.50 | 0.51 | 0.50 | 0.51 |
| **NRL** | 0.57 | 0.56 | 0.63 | 0.59 | 0.59 | 0.60 |
| **FSW** | 0.30 | 0.30 | 0.35 | 0.30 | 0.31 | 0.34 |
| **DSW** | 0.36 | 0.38 | 0.40 | 0.40 | 0.41 | 0.39 |
| **LA** | 0.42 | 0.42 | 0.44 | 0.43 | 0.46 | 0.48 |

BL = Bayesian LASSO; BRR = Bayesian ridge regression
