# Supplementary material for: Genome-wide association study and genomic prediction of root system architecture traits in Sorghum (Sorghum bicolor (L.) Moench) at the seedling stage
Source: BMC Plant Biol. 2025 Jan 17;25:69. doi: 10.1186/s12870-025-06077-w (PMC11740658; doi:10.1186/s12870-025-06077-w)
Supplement: Supplementary file 6 — Supplementary Material 6: Supplementary Table 6: Summary of previously identified SNPs associated with the root system architecture (RSA) traits. [file 12870_2025_6077_MOESM6_ESM.docx]

**Supplementary Table 6.** Summary of previously identified SNPs associated with RSA traits.

| **Trait** | **Population** | **No of samples** | **No of markers** | **Type of Markers** | **Chr/LG** | **Position** | **Method** | **Reference** |
| --- | --- | --- | --- | --- | --- | --- | --- | --- |
| NRA | Ethiopian sorghum accessions | 274 | 265,944 | SNP | 1 | 56872999 | GWAS | [1] |
|  |  |  |  |  | 2 | 47979513 |  |  |
|  |  |  |  |  | 4 | 6185121 |  |  |
|  |  |  |  |  | 6 | 48244728 |  |  |
|  |  |  |  |  | 7 | 48851050 |  |  |
|  |  |  |  |  | 9 | 1212069 |  |  |
|  | Sorghum accessions | 129 | 127,804 | SNP | 1 | 53254066 | GWAS | [2] |
|  |  |  |  |  | 3 | 18779995 |  |  |
|  |  |  |  |  | 4 | 56610955 |  |  |
|  |  |  |  |  | 6 | 2724950 |  |  |
|  |  |  |  |  | 9 | 48742385 |  |  |
|  | Ethiopian sorghum landraces | 940 | 54,080 | SNP | 3 | 70640280 | GWAS | [3] |
|  |  |  |  |  | 4 | 52404340 |  |  |
|  |  |  |  |  | 5 | 7621214 |  |  |
|  |  |  |  |  | 6 | 47152373 |  |  |
|  |  |  |  |  | 6 | 49330161 |  |  |
|  |  |  |  |  | 7 | 53856839 |  |  |
|  |  |  |  |  | 8 | 47689441 |  |  |
|  | RILs | 155 | 337 | DArT | 5 | 51.8 | LM | [4] |
|  |  |  |  |  | 5 | 34 |  |  |
|  |  |  |  |  | 8 | 25.4 |  |  |
|  |  |  |  |  | 10 | 208.4 |  |  |
|  | F3:4 and F4:5Mapping population | 135 | 282,267 | SNP | 3 | 4757527 | LM | [5] |
| NRN | Ethiopian sorghum accessions | 274 | 265,944 | SNP | 1 | 56872999 | GWAS | [1] |
|  |  |  |  |  | 2 | 47979513 | GWAS |  |
|  |  |  |  |  | 4 | 6185121 | GWAS |  |
|  |  |  |  |  | 6 | 48244728 | GWAS |  |
|  |  |  |  |  | 7 | 48851050 | GWAS |  |
|  |  |  |  |  | 9 | 1212069 | GWAS |  |
|  | Ethiopian sorghum landraces | 940 | 54,080 | SNP | 1 | 76107179 | GWAS | [3] |
|  |  |  |  |  | 2 | 19789147 | GWAS |  |
|  |  |  |  |  | 2 | 60605417 | GWAS |  |
|  |  |  |  |  | 2 | 73281144 | GWAS |  |
|  |  |  |  |  | 6 | 5,403226 | GWAS |  |
|  |  |  |  |  | 8 | 53766348 | GWAS |  |
|  |  |  |  |  | 9 | 49867350 | GWAS |  |
|  | F8 RILs (E36-1 ×SPV70) | 184 | 104 | EST-SSR, SSR, SNP | 1 | 181.51 | LM | [6] |
|  | Sorghum accessions | 129 | 127,804 | SNP | 1 | 6012717 | LM | [2] |
| NRL | Ethiopian sorghum accessions | 274 | 265,944 | SNP | 2 | 58681356 | GWAS | [1] |
|  |  |  |  |  | 5 | 65538924 |  |  |
|  | Diverse sorghum accessions | 96 | 43,452 | SNP | 1 | 54515547 | GWAS | [7] |
|  |  |  |  |  | 5 | 18563597 |  |  |
|  | Ethiopian sorghum landraces | 940 | 54,080 | SNP | 1 | 66615979 | GWAS | [3] |
|  |  |  |  |  | 3 | 2540858 |  |  |
|  |  |  |  |  | 3 | 10025278 |  |  |
|  |  |  |  |  | 5 | 53377135 |  |  |
|  | Sorghum accessions | 129 | 127,804 | SNP | 1 | 6012717 | GWAS | [2] |
|  |  |  |  |  | 4 | 13304368 |  |  |
|  |  |  |  |  | 4 | 57625836 |  |  |
|  |  |  |  |  | 4 | 60963038 |  |  |
|  |  |  |  |  | 6 | 45176048 |  |  |
|  | F8 RILs (E36-1 ×SPV70) | 184 | 104 | EST-SSR, SSR, SNP | 4 | 32.21 | LM | [6] |
|  | sorghum accessions from the U.S. sorghum association panel | 300 | 265,487 | SNPs | 2 | 56602226 | GWAS | [8] |
|  |  |  |  |  | 2 | 54575799 |  |  |
|  |  |  |  |  | 2 | 54593968 |  |  |
|  |  |  |  |  | 2 | 54593969 |  |  |
|  |  |  |  |  | 2 | 56566217 |  |  |
|  |  |  |  |  | 2 | 67807062 |  |  |
|  |  |  |  |  | 2 | 72583772 |  |  |
|  |  |  |  |  | 2 | 72833569 |  |  |
|  |  |  |  |  | 2 | 73739699 |  |  |
|  |  |  |  |  | 3 | 16201529 |  |  |
|  |  |  |  |  | 4 | 60946340 |  |  |
|  |  |  |  |  | 5 | 1302419 |  |  |
|  |  |  |  |  | 9 | 8614594 |  |  |
| SDW | Sorghum association panel | 272 | 295,914 | SNPs | 2 | 4581950 | GWAS | [7] |
|  |  |  |  |  | 2 | 63000541 |  |  |
|  |  |  |  |  | 3 | 27729075 |  |  |
|  |  |  |  |  | 4 | 66063042 |  |  |
|  |  |  |  |  | 9 | 56525300 |  |  |
|  |  |  |  |  | 10 | 52722710 |  |  |
|  | RILs | 155 | 337 | DArT | 1 | 254.2 | LM | [4] |
|  |  |  |  |  | 5 | 107.7 |  |  |
|  | Sorghum accessions | 129 | 127,804 | SNPs | 7 | 64332829 | GWAS | [2] |
| SFW | sorghum accessions from the U.S. sorghum association panel | 300 | 265,487 | SNPs | 3 | 47228001 | GWAS | [8] |
|  |  |  |  |  | 6 | 57163269 | GWAS |  |
|  |  |  |  |  | 6 | 57302179 |  |  |
|  |  |  |  |  | 6 | 57163127 |  |  |
|  |  |  |  |  | 6 | 57170126 |  |  |
| LA | Sorghum association panel | 272 | 295,914 | SNPs | 2 | 72741053 | GWAS | [9] |
|  |  |  |  |  | 4 | 12354434 |  |  |
|  |  |  |  |  | 7 | 11107766 |  |  |
|  |  |  |  |  | 9 | 55485561 |  |  |
|  | RILs | 185 | 337 | DArT | 8 | 104 | LM | [4] |
|  |  |  |  |  | 8 | 123.3 |  |  |
|  |  |  |  |  | 8 | 237.6 |  |  |
|  | Sorghum accessions | 129 | 127,804 | SNP | 1 | 68144065 | GWAS | [2] |
|  |  |  |  |  | 6 | 49860654 |  |  |
|  |  |  |  |  | 6 | 6049929 |  |  |

**References**

1. Elias M, Chere D, Lule D, Serba D, Tirfessa A, Gelmesa D, Tesso T, Bantte K, Menamo TM. Multi‐locus genome‐wide association study reveal genomic regions underlying root system architecture traits in Ethiopian sorghum germplasm. TPG 2024:e20436.

2. Kebede A, Barka GD, Kebede M, Tadesse T, Girma G, Menamo TM. Multi-locus genome-wide association analysis for root and shoot traits at seedling stage in Ethiopian sorghum (*Sorghum bicolor* (L.) Moench) accessions. Genet. Resour. Crop Evol. 2024:1-23.

3. Menamo T, Borrell AK, Mace E, Jordan DR, Tao Y, Hunt C, Kassahun B. Genetic dissection of root architecture in Ethiopian sorghum landraces. Theor. Appl. Genet. 2023; 136(10):209.

4. Mace E, Singh V, Van Oosterom E, Hammer G, Hunt C, Jordan D. QTL for nodal root angle in sorghum (Sorghum bicolor L. Moench) co-locate with QTL for traits associated with drought adaptation. Theor. Appl. Genet. 2012; 124(1):97-109.

5. Lopez JR, Erickson JE, Munoz P, Saballos A, Felderhoff TJ, Vermerris W. QTLs associated with crown root angle, stomatal conductance, and maturity in Sorghum. TPG 2017; 10(2):plantgenome2016.2004.0038.

6. Rajkumar, Fakrudin B, Kavil S, Girma Y, Arun S, Dadakhalandar D, Gurusiddesh B, Patil A, Thudi M, Bhairappanavar S. Molecular mapping of genomic regions harbouring QTLs for root and yield traits in sorghum (Sorghum bicolor L. Moench). Physiol. Mol. Biol. Plants 2013; 19:409-419.

7. Ramalingam AP, Mohanavel W, Kambale R, Rajagopalan VR, Marla SR, Prasad PV, Muthurajan R, Perumal R. Pilot-scale genome-wide association mapping in diverse sorghum germplasms identified novel genetic loci linked to major agronomic, root and stomatal traits. Scientific Reports 2023; 13(1):21917.

8. Chopra R, Burow G, Burke JJ, Gladman N, Xin Z. Genome-wide association analysis of seedling traits in diverse Sorghum germplasm under thermal stress. BMC Plant Biol. 2017; 17:1-15.

9. Hufnagel B, Bernardino KC, Malosetti M, Sousa SM, Silva LA, Guimaraes CT, Coelho AM, Santos TT, Viana JH, Schaffert RE. Multi-trait association mapping for phosphorous efficiency reveals flexible root architectures in sorghum. BMC Plant Biol. 2024; 24(1):562.
